# Diarrhœa of Consumptives

**Published:** 1889-05

**Authors:** F. L. Griffith

**Affiliations:** Edina, Mo.


### DIARRHŒA OF CONSUMPTIVES.

F. L. GRIFFITH, M. D., EDINA, MO.

Mrs. ——, age twenty-two, in the last stage of consumption, was attacked in June last with a most weakening diarrhœa. She had been under the care of allopaths for several months, till last August, when I was called. Her friends knew the case was hopeless and expected her to die in the fall, but they were very desirious of having the troublesome and weakening diarrhœa stopped. I gave Sulph.$^{cm}$, Podo.$^{200}$, Lyc.$^{cm}$. I gave these three drugs from August to October, one dose of each about a month apart, without effect, yet they each at various times seemed indicated. About the middle of October they were expecting her to die every day, and I had given up all hopes, till one day in carefully looking over my interleaved Lippe, I noticed a line, viz.: "Diarrhœa of consumptives—Acetic acid." I determined immediately to try it. I gave three powders of the 30cm potency, to be taken dry, one each night, till the three were used; it acted marvelously; it not only stopped the diarrhœa, but gave her a natural and regular action. The patient got stronger and lived till February 15th. She was not troubled any more till a couple of days before her death. I had copied the idea from Dr. Kent's interleaved repertory, and have proved it in one case most effectually. I could not see, in the provings of Acet-ac., anything similar to my case, but it most certainly seems to act in this disease where the indicated remedy fails.

